# Ultrasound measures of muscle thickness may be superior to strength testing in adults with knee osteoarthritis: a cross-sectional study

**DOI:** 10.1186/s12891-018-2267-4

**Published:** 2018-09-27

**Authors:** Alfred C. Gellhorn, Jordan M. Stumph, Hashem E. Zikry, Carly A. Creelman, Rachel Welbel

**Affiliations:** 1 Department of Rehabilitation Medicine, Weill Cornell Medicine, 525 E 68th Street, B16, New York, NY 10065 USA; 2 Albert Einstein College of Medicine, New York, NY USA; 3 Icahn School of Medicine at Mount Sinai, New York, NY USA

**Keywords:** Ultrasound, Osteoarthritis, Reliability, Strength

## Abstract

**Background:**

Evaluation of muscle strength as performed routinely with a dynamometer may be limited by important factors such as pain during muscle contraction. Few studies have compared formal strength testing with ultrasound to measure muscle bulk in adults with knee osteoarthritis (OA).

**Methods:**

We investigated the muscle bulk of lower limb muscles in adults with knee OA using quantitative ultrasound. We analyzed the relationship between patient reported function and the muscle bulk of hip adductors, hip abductors, knee extensors and ankle plantarflexors. We further correlated muscle bulk measures with joint torques calculated with a hand held dynamometer. We hypothesized that ultrasound muscle bulk would have high levels of interrater reliability and correlate more strongly with pain and function than strength measured by a dynamometer. 23 subjects with unilateral symptomatic knee OA completed baseline questionnaires including the Western Ontario and McMaster Universities Arthritis Index (WOMAC) and Lower Extremity Activity Scale. Joint torque was measured with a dynamometer and muscle bulk was assessed with ultrasound.

**Results:**

Higher ultrasound measured muscle bulk was correlated with less pain in all muscle groups. When comparing muscle bulk and torque measures, ultrasound-measured muscle bulk of the quadriceps was more strongly correlated with measures of pain and function than quadriceps isometric strength measured with a dynamometer.

**Conclusions:**

Ultrasound is a feasible method to assess muscle bulk of lower limb muscles in adults with knee OA, with high levels of interrater reliability, and correlates negatively with patient reported function. Compared with use of a hand held dynamometer to measure muscle function, ultrasound may be a superior modality.

## Background

Osteoarthritis (OA) is the most prevalent joint disease in the United States, with high levels of pain and functional disability in individuals affected by the disease. OA of the knee is particularly problematic, with the lifetime risk of developing knee OA estimated at 47% among women and 40% among men [1]. Conservative management strategies for knee OA frequently include therapeutic exercise, often with the guidance of a physical therapist to direct the specific exercise program. Muscular strength and neuromuscular control may modulate joint forces and this premise forms the basis for many physical therapy interventions in OA. Despite generally positive results from trials evaluating therapeutic exercise in adults with knee OA, there remains a lack of understanding about which muscle groups are most important in modifying joint forces, and, indeed, whether improvement in strength is the reason for the positive outcomes seen after such interventions.

Joint forces are due to the bulk and composition of various muscle groups, the associated lever arm, and neural activation patterns that activate groups of muscles to produce joint motion. Measured strength as performed routinely with a dynamometer may be a useful indicator of the ability of muscle to affect force production upon a joint, but when tested at pathologic joints may be limited by important factors such as pain during muscle contraction. Pure muscle mass is another way to measure the theoretical ability of muscle to generate force; in situations where there is no pain during movement, muscle physiologic cross sectional area correlates strongly with muscle force generation [2]. Ultrasound has emerged as a safe and reliable method to evaluate muscle thickness, and these measurements correlate with muscle cross sectional area, [3] suggesting that ultrasound-measured muscle thickness may provide important information about muscle function.

While it is established that quadriceps muscle strength influences pain and function in knee OA, it is unknown whether similar associations exist for muscles at the hips and the ankles. Theoretically, as the primary knee extensors, the quadriceps are important in force modulation: the quadriceps are highly active during the majority of the gait cycle and slow the rapid knee flexion produced during initial contact when knee joint forces and the rate of loading are highest [4]. Hip abductors and hip adductors are also theoretically important given their role in controlling the position of the limb during gait. By determining the degree of limb adduction or abduction, these muscles will influence the ground reaction force vector relative to the center of the knee joint in the coronal plane [4]. Finally, the plantar flexors are important in many models of gait, and peak plantar flexor moments in adults with knee OA predict knee joint compressive forces [5]. Because of the possible importance of all of these muscle groups in influencing forces across the knee, an understanding of the relative importance of each muscle group on symptom generation would represent a positive advance.

Our primary aim in this preliminary study is to investigate the relationship between ultrasound measured bulk of the hip, knee, and ankle muscles and self-reported function in adults with knee OA. Secondarily, we aim to compare these relationships with strength as measured more conventionally using a hand held dynamometer.

## Methods

### Subjects and data collection

Subjects in this study included 23 adults with unilateral symptomatic knee osteoarthritis, recruited from the outpatient clinic of the primary investigator. Knee OA was diagnosed using American College of Rheumatology (ACR) guidelines [6] based on clinical and radiographic findings. All subjects were screened by telephone for their suitability for enrollment based on ACR guidelines including pain in the knee and at least one of the following: age greater than 50 years, morning stiffness less than 30 min, and joint crepitus. Subjects were excluded from the study if they had any of the following: a prior corticosteroid injection into the knee within 4 weeks prior to enrollment, a prior diagnosis of a neuromuscular condition that affected lower extremity strength, or an alternative rheumatologic diagnosis explaining their knee pain. If subjects met these criteria, they received a weight bearing anterior-posterior and lateral radiograph of both knees. Based on ACR guidelines, the presence of osteophytes on the symptomatic knee was required for radiographic diagnosis of OA. Once subjects met clinical and radiographic inclusion and exclusion criteria, they were entered into the study. Data were collected by trained research assistants in a single in-person visit. The study was approved by the host institution’s IRB and all patients provided written informed consent.

### Variables

The Western Ontario and McMaster Universities Arthritis Index (WOMAC) was used to assess subjects’ pain, stiffness, and physical functioning. The WOMAC questionnaire is well validated in adults with knee OA and includes 24 questions that measures the three dimensions of pain, disability and joint stiffness.

The Lower Extremity Activity Scale (LEAS) [7] was used to determine the level of daily physical activity in each patient. The LEAS is a self-administered 18-level questionnaire that has been validated in adults with knee OA.

Anthropomorphic measurements, including height and weight, were obtained to calculate joint torques and normalize muscle thickness measurements. Length of the lower limb was measured from the anterior superior iliac spine (ASIS) to the lateral malleolus, and the lower leg was measured from the lateral femoral condyle to the lateral malleolus. All lower limb measurements were performed by a trained research assistant with the subject supine using a flexible tape measure. The ASIS, lateral femoral condyle, and lateral malleolus were identified by palpation. The average of two separate measures was used for the calculating limb length based on previous reports of optimizing validity of this measurement method [8].

Kellgren Lawrence grading of the radiographic degree of osteoarthritis was performed for both knees by the primary investigator.

### Ultrasound measurements

Muscle groups evaluated with ultrasound imaging included the knee extensor group (quadriceps femoris); hip abductor group (gluteus medius and minimus); hip adductor group (adductor brevis, adductor longus, adductor magnus, and gracilis); and ankle plantarflexor group (gastrocnemius and soleus). Prior to obtaining ultrasound measures on study participants, we developed a standardized protocol for measuring muscle thickness using normal volunteers to ensure maximal interrater reliability. Two evaluators were trained to perform ultrasound scans following the same protocol. For each muscle studied, we used bony landmarks and surface markings to identify a location as close as possible to the mid-portion of the muscle belly. For the quadriceps and hip adductors, a skin mark was placed at half of the distance between the greater trochanter and the lateral condyle of the femur. This line was extended circumferentially across the anterior and medial leg to obtain consistent imaging of the quadriceps and adductors. Next, a mark was placed at 30% from the distal end of a line between the lateral femoral condyle and the lateral malleolus at the ankle. This corresponded to the mid-portion of the gastrocnemius and soleus. A final mark was placed at half the distance from the ASIS to the greater trochanter of the femur, corresponding to the mid-portion of the gluteus medius and minimus.

**Fig. 1:**
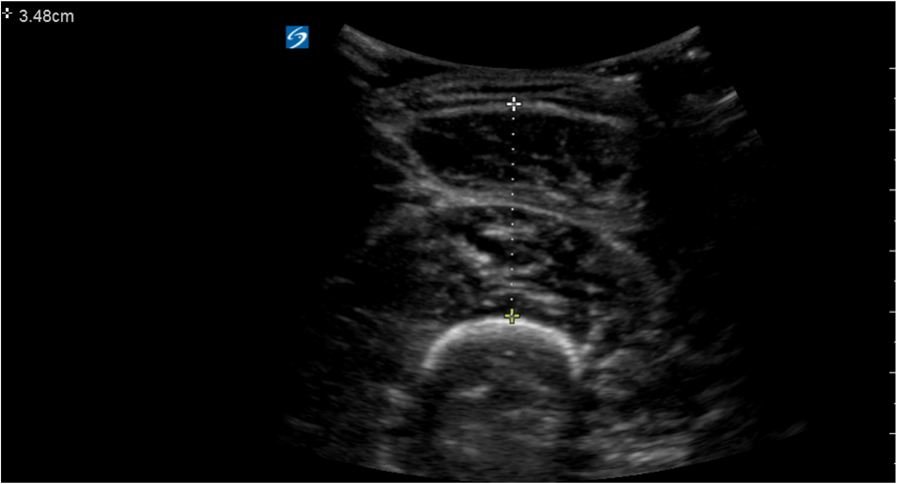
Ultrasound image of the quadriceps, measured at mid thigh. Calipers demarcate the muscle thickness, measured from the perimysium of the rectus femoris to the cortex of the femur

A Sonosite X-Porte (Bothell, WA) with a curvilinear 5–2 MHz transducer was used to obtain all ultrasound images. Subjects lay supine on an exam table. The transducer was placed perpendicular to the skin/musculature to minimize risk of sampling a muscle obliquely and to ensure repeatability. After the muscle was identified, the examiner slightly retracted the transducer so as to not compress the muscle; the image was considered to be optimized when a thin film of gel was present between the skin and the transducer indicating that no manual compressive forces were distorting the muscle. Once the ultrasound image was optimized, a still image was captured and the muscle thickness was measured with caliper-based tools included in the machine software (Fig. 1). The process was repeated three times for each muscle group and all three measurements were recorded. Once all images were obtained from one lower extremity, the same method was used for imaging of the other.

### Strength measurements

A Lafayette Model 01165 hand-held dynamometer (Lafayette, IN) was used to measure peak force over a 3 s period, as per settings on the dynamometer. Anatomical markers were used for dynamometer placement to achieve accurate lever arm measurements. When obtaining measurements for the hip abductors, the subject was placed in the supine position, and the dynamometer was placed 5 cm proximal to the lateral malleolus on the lateral side of the lower leg. The subject was cued to abduct the leg against the resisted pressure of the dynamometer. For the adductors, the subject was again supine, and the dynamometer was placed 5 cm proximal to the medial malleolus on the medial aspect of the lower leg, and instructed to adduct the leg against the resisted pressure of the dynamometer. Finally, for the quadriceps, the subject was seated and the dynamometer was placed in the midline at 5 cm proximal to the lateral malleolus. We chose these locations based on prior studies that indicated high levels of reliability and validity [9–11].

All of our strength tests were isometric “make tests”, such that the subject pushed against the dynamometer while the examiner maintained the dynamometer as steadily as possible. For each test, the subject was allowed to have one warm-up (~ 50% maximum strength) to account for any habituation. The test was repeated three times for each muscle group. Each subject was given a 30 s rest period after each of the tests performed to avoid fatiguing the subject. All tests lasted 3 s as determined by the dynamometer. The settings on the machine itself were set to stop recording with an audible beep after this time period had elapsed. To initiate each test, the subjects were instructed to “go” then the examiner repeated “push, push, push” to signal the patient to push as hard as possible for the remaining 3 s of the test. After the dynamometer beeped, the examiner told the subject to “relax” to signal the end of the test. Maximal force attained during each attempt was recorded.

Based on prior studies regarding the ideal method of reporting strength in knee OA, we calculated joint torque as the product of the force measured by the dynamometer and the distance from the dynamometer to the axis of rotation of the joint [4]. Additionally, because strength varies with body size in adults with and without OA, [12] we calculated strength relative to body mass in kg.

### Analysis

All analysis was performed using Microsoft Excel 15.1 (Redmond, WA) and STATA 14.1 (College Station, TX), with alpha level for hypothesis testing set at 0.05. Torque was calculated at each joint by multiplying the force obtained by dynamometry by the lever arm of the limb. For instance, knee extensor torque was calculated by multiplying the strength of knee extension by the length of the lower leg, and is reported in units of Newton meters (Nm).

Data were evaluated for normality using the Shapiro Wilk test and normal quantile plots. We used simple descriptive statistics to describe our cohort, and paired t-tests to evaluate for any differences in muscle parameters between symptomatic and asymptomatic limbs. Because some of the strength measures were not normally distributed, we used Spearman’s rho to evaluate the correlation between baseline characteristics and muscle measures as well as between functional measures and muscle parameters. We considered r values < 0.3 to represent a weak association, 0.3–0.7 to represent a moderate association, and > 0.7 to represent a strong association [13].

To evaluate the relationship between muscle measures and WOMAC in more detail, we performed a simple linear regression analysis, with the total WOMAC score as the dependent variable, and muscle thickness or torque as the independent variable. To control for possible confounding, we performed a multivariable linear regression analysis using age and gender as covariates. We chose age and gender as possible confounders based on the conceptual model that muscle bulk and strength are correlated with both of these variables. In the multivariable analysis, we assessed how much the regression coefficient associated with the muscle measure changed after adjusting for each potential confounder. If the regression coefficient from the simple linear regression model changed by more than 10%, then the covariate was felt to represent a confounder, and was included in the final regression model [14].

To determine the reliability of measurements for both ultrasound thickness and muscle force, we calculated intra-class correlation coefficients (ICCs) (2,1), using a two-way mixed effects model [15]. ICC (2,1) was used because we were interested in generalizing findings beyond the two raters in the study. An ICC > 0.75 was considered good and ICC > 0.9 was considered excellent [16].

## Results

### Subject characteristics

Subject baseline characteristics are shown in Table 1. Subjects included 12 females and 11 males with average age of 63.8 years. The majority of patients had moderate osteoarthritis based on the Kellgren Lawrence scale, with chronic painful symptoms due to OA and median symptom duration of 2 years. No subjects had grade 4 radiographic osteoarthritis. Some patients had radiographic osteoarthritis on the contralateral, asymptomatic knee, though radiographic osteoarthritis grade was less on the asymptomatic side. Symptoms as measured by the WOMAC index were mild to moderate, with a mean total WOMAC score of 25, on a scale from 0 to 96, where higher scores indicate worse symptoms. Functional daily activity as measured by the LEAS had a mean score of 13.1, on a scale of 1–18, where higher scores relate to greater daily functional activity.

**Table 1:** Subject baseline characteristics, *N* = 23

	Mean (SD) or percent
Age	63.8 (9.3)
Gender, female	52%
Weight, kg	77.4 (14.5)
BMI	26.9 (3.7)
Pain level	4.1 (1.8)
Symptomatic side, right	52%
Symptom duration (months)	44.8 (62.1)
Symptomatic KL grade	
0	0
1	1
2	9
3	12
4	0
Asymptomatic KL grade	
0	8
1	7
2	7
3	0
4	0
WOMAC pain subscale (0–20)	4.6 (3.2)
WOMAC stiffness subscale (0–8)	3.1 (1.7)
WOMAC function subscale (0–68)	17.45 (13.3)
WOMAC total (0–96)	25.3 (17.4)

*NRS* Numeric Rating System, *BMI* Body Mass Index, *KL* Kellgren Lawrence, *WOMAC* Western Ontario and McMaster Arthritis Index

### Strength and muscle bulk measurements

Subject muscle characteristics are presented in Table 2. There were no significant differences in normalized measured strength (Nm/kg) between symptomatic and asymptomatic limbs. Similarly, there were no differences in muscle bulk of any of the investigated muscles between symptomatic and asymptomatic limbs.

**Table 2:** Subject muscle characteristics

			Paired t-test
	Symptomatic	Asymptomatic	
Strength measured as torque (Nm) normalized to body weight (kg)			
Knee extensor	96 (58.9)	95.2 (53.6)	0.84
Hip abductors	85.8 (27.1)	88.5 (29.1)	0.44
Hip adductors	90.4 (31.8)	91.9 (34.2)	0.72
Ankle plantarflexors	29.1 (11.1)	30.3 (11.7)	0.26
Muscle thickness (mm) normalized to weight (kg)			
Quadriceps	0.37 (0.12)	0.38 (0.12)	0.32
Hip abductors	0.42 (0.13)	0.42 (0.13)	0.72
Hip adductors	0.59 (0.18)	0.60 (0.18)	0.24
Ankle plantarflexors	0.56 (0.24)	0.56 (0.22)	0.98
Muscle thickness (mm) non-normalized			
Quad	28.4 (9.1)	29.2 (9.3)	0.27
Hip abductors	32.0 (10.9)	31.7 (10.2)	0.61
Hip adductors	44.7 (13.3)	45.4 (12.5)	0.34
Ankle plantarflexors	42.4 (17.5)	42.4 (16.2)	0.99

The terms in parentheses indicate standard deviations

### Inter-rater reliability of ultrasound and strength measures

Intraclass correlation coefficients (ICCs) for ultrasound measurements were excellent for all ultrasound measures. ICC (2,1) was 0.95 for quadriceps, 0.92 for hip adductors, 0.91 for hip abductors, and 0.98 for ankle plantarflexors. ICC(2,1) for torque at the hip adductors was excellent (0.93), but only good at quadriceps (0.83), hip abductors (0.87), and ankle plantarflexors (0.77). Reliability was markedly better for ultrasound measures than torque measures at the quadriceps, hip abductors and ankle plantarflexors.

### Correlations between baseline characteristics, muscle characteristics, and functional measures

Female gender was moderately associated with higher pain as measured by the WOMAC pain sub-scale. No other correlations between baseline characteristics and WOMAC or LEAS scales reached statistical significance.

### Correlation of function, pain and muscle measures

Muscle bulk correlated negatively with pain scores such that greater muscle bulk was associated with lower pain scores (Table 3). This association was significant for the quadriceps and hip adductors but did not reach significance in other muscle groups. Quadriceps thickness was strongly correlated with function, with greater thickness associated with better function. Other muscle groups showed mild to moderate correlation with function, with significance seen in the symptomatic hip adductors. Symptomatic joint stiffness was not found to correlate with any measured muscle thickness. Age and symptom duration were not correlated with muscle thickness in any muscle groups. Males showed higher values for muscle thickness than females for all muscle groups.

**Table 3:** Unadjusted Spearman’s rho correlations between muscle measures and functional measures

Muscle group	WOMAC pain	WOMAC stiffness	WOMAC function	WOMAC total	Age	Gender	BMI	Symptom duration
Muscle strength measures								
Symptomatic knee extension	−0.42	− 0.18	− 0.35	− 0.36	− 0.10	0.49 *	0.18	0.21
Asymptomatic knee extension	− 0.31	− 0.17	− 0.29	− 0.27	− 0.02	0.41	− 0.20	0.20
Symptomatic hip abduction	− 0.52 *	− 0.22	− 0.46 *	− 0.47 *	− 0.25	0.17	0.01	0.11
Asymptomatic hip abduction	− 0.49 *	− 0.28	− 0.51 *	−0.51 *	− 0.22	0.47 *	0.04	0.10
Symptomatic hip adduction	−0.52 *	−0.13	− 0.44 *	−0.44 *	0.02	0.36	−0.09	0.07
Asymptomatic hip adduction	−0.51 *	−0.25	− 0.54 *	**−0.54** ‡	0.10	0.28	−0.07	−0.08
Symptomatic ankle plantarflexion	−0.46 *	−0.06	− 0.49 *	−0.47 *	− 0.43 *	0.30	− 0.09	0.19
Asymptomatic ankle plantarflexion	−0.46 *	−0.18	−.0.42 *	− 0.42 *	−0.35	0.25	−0.04	0.27
Muscle thickness measures								
Symptomatic quadriceps thickness	−0.48 *	−0.09	**− 0.62 ‡**	**−0.60 ‡**	− 0.35	0.37	− 0.04	0.15
Asymptomatic quadriceps thickness	−0.38	−0.22	− 0.54 *	−0.53 *	− 0.29	0.51 *	− 0.01	0.15
Symptomatic hip abductor thickness	−0.13	−0.09	− 0.14	−0.14	0.05	0.41 *	0.03	−0.10
Asymptomatic hip abductor thickness	−0.20	− 0.02	− 0.25	− 0.22	− 0.05	0.43 *	0.08	−0.10
Symptomatic hip adductor thickness	−0.45 *	− 0.11	− 0.47 *	−0.44 *	− 0.02	0.64 *	− 0.30	− 0.06
Asymptomatic hip adductor thickness	−0.45 *	− 0.10	− 0.40	−0.38	− 0.04	**0.58 ‡**	− 0.27	− 0.11
Symptomatic calf thickness	− 0.39	− 0.04	− 0.39	−0.37	− 0.14	0.49 *	− 0.12	−0.05
Asymptomatic calf thickness	−0.37	−0.03	− 0.37	−0.35	− 0.15	0.47 *	− 0.14	−0.08

*WOMAC* Western Ontario and McMaster Arthritis Index, *BMI* Body Mass Index

Strength measured in torque (Nm) normalized to body weight (kg), ie Nm/kg

Values indicated by * with bold text indicates significance at 0.05 level, ‡ with bold text indicates significance at 0.01 level

Similar to ultrasound-measured bulk, muscle torque generated by all muscle groups was negatively correlated with pain such that lower muscle torque was correlated with worse pain (Table 3). This correlation reached levels of significance for hip abductors, hip adductors, and plantarflexors on both limbs. Importantly, there was no significant correlation found between pain and quadriceps torque. Analyzing correlation with function, muscle torques were negatively correlated with function, with significant correlation seen in the hip abductors, adductors, and plantarflexors, but not quadriceps.

### Regression analysis

In the simple linear regression analysis, quadriceps thickness was the only ultrasound measure significantly associated with the total WOMAC score. Conversely, dynamometer-measured strength of the quadriceps was not significantly associated with total WOMAC score, while strength of the abductors, adductors, and plantarflexors did show a significant association. When assessing for confounding by age and gender in the multivariable model, age did not change the regression coefficient by more than 10% for any of the strength or muscle thickness measures and was therefore deemed not a confounder. On the other hand, the addition of gender to the model resulted in a change in the regression coefficient by more than 10%, and so was considered a confounder and included in the final regression model. The full results of the multivariable regression analysis are presented in Table 4. In the final model, the unadjusted beta for symptomatic quadriceps thickness normalized to weight was − 67.2. In other words, for every 1 mm/kg increase in quadriceps thickness, the corresponding total WOMAC score decreased by 67.2. To place this in context, we calculated the minimum clinically important difference in WOMAC for this group as a 10% change in the mean WOMAC score, or 2.4 points. Using the above unadjusted beta, for a 70 kg adult, an increase in quadriceps thickness of 2.4 mm would be associated with an improvement of 2.4 on the WOMAC scale.

**Table 4:** Summary of multivariable regression analysis for muscle characteristics predicting the total WOMAC score, controlled for gender

Predictor	Unadjusted beta	*p*-value
Muscle thickness measures (mm/kg)		
Symptomatic quadriceps	−67.2	0.009 *
Asymptomatic quadriceps	−60.5	0.031 *
Symptomatic hip abductors	− 11	0.66
Asymptomatic hip abductors	−21.7	0.85
Sympatomatic hip adductors	−27.8	0.13
Asympatomatic hip adductors	−29	11
Symptomatic calf	−20.6	0.14
Asymptomatic calf	−19.9	0.19
Muscle torque measures (Nm/kg)		
Symptomatic knee extensors	−0.036	0.57
Asymptomatic knee extensors	−0.051	0.12
Symptomatic hip abductors	−0.197	0.08
Asymptomatic hip abductors	−0.138	0.27
Symptomatic hip adductors	−0.126	0.26
Asymptomatic hip addutors	−0.188	0.06
Symptomatic ankle plantarflexors	−0.65	0.05
Asymptomatic ankle plantarflexors	−0.56	0.05

* indicates *p* < 0.05

## Discussion

This exploratory study identified a number of muscle characteristics that were associated with measures of pain and function in adults with knee OA. However, it is notable that muscle torque and ultrasound-measured muscle bulk did not always demonstrate the same degree of correlation with pain and function. Most notably, while quadriceps muscle bulk was strongly correlated with the WOMAC functional subscale and overall WOMAC score, quadriceps torque was not. This suggests that for some muscle groups, measuring torque alone may give an inadequate picture of the muscle’s functional ability. In other words, muscle strength and muscle bulk do not provide the same information in adults with painful knee OA.

The divergence we observed between muscle torque and muscle bulk is not entirely surprising, since control at a joint is due to neural activation patterns as well as muscle bulk and fat infiltration. Neural activation patterns, in particular, are likely altered when activation of the muscle compresses a painful joint. Arthrogenic muscle inhibition is well described in painful knees, [17] wherein afferent discharge from neurons that innervate the knee joint have effects on spinal and supraspinal pathways to limit activation of the quadriceps muscle.

Therefore, measurement of quadriceps strength alone, as performed in many prior studies evaluating function in adults with knee OA [18–23] may provide an incomplete picture of the role of the quadriceps in predicting function. Indeed, a number of studies have attempted to account for the possibility of arthrogenic muscle inhibition using test techniques such as burst-superimposition, where electrical stimulation of muscle is superimposed on a muscle undergoing active contraction [23, 24]. While theoretically attractive, this type of testing is complex and painful.

We propose that ultrasound measured muscle bulk provides a complimentary method of determining muscle function in adults with knee OA, and our findings that quadriceps muscle thickness correlates significantly with function and overall WOMAC score supports this premise. The idea of an imaging biomarker that correlates with functional and pain measures is attractive and minimizes many of the above concerns about isometric strength testing to measure muscle function. Supporting this, a recent study showed that MRI measured change of quadriceps cross sectional area was both more sensitive to longitudinal change and correlated more strongly with disease progression when compared with isometric strength testing in a large cohort of patients with symptomatic knee OA [25]. While the costs and logistics of MRI preclude its use in a clinical setting to assess muscle function, ultrasound provides an appealing alternative that is likely feasible for most clinical and research settings.

Our use of quantitative ultrasound analysis to measure muscle bulk is based on data showing high levels of inter-rater, intra-rater, and inter-machine reliability when using a well described scanning protocol [26]. Furthermore, a strict scanning protocol enables even a novice ultrasound practitioner to achieve high levels of reliability with minimal training [26, 27], increasing the applicability of this technique. Importantly, our study had excellent levels of inter-rater reliability for all ultrasound measures, and were significantly better than measures of torque for the quadriceps, hip abductors and ankle plantarflexors. The ultrasound examination itself is well tolerated and rapid, with acquisition of images taking approximately 5 min, and measurement taking an additional 5–10 min, depending on the software included on the ultrasound unit.

By evaluating multiple muscle groups at once in the same subjects, we aimed to describe the relative importance of muscle strength at the knee, hip, and ankle in moderating symptoms of knee OA. A picture emerges of a beneficial effect of greater muscle strength in all muscle groups measured, though our data show that the strongest association between muscle function and symptoms is seen with the quadriceps. This is in line with many prior studies that have shown the importance of quadriceps strength [4] and that form the basis for many therapeutic exercise interventions. However, our data suggest that muscle evaluation and therapy should not be limited to quadriceps alone, and that the hip adductors, hip abductors, and ankle plantarflexors all contribute to improved lower limb function.

While we found moderate to strong correlations between muscle strength and WOMAC pain and function scales, we found no similar correlation between muscle strength and WOMAC stiffness subscale. While the etiology of symptomatic joint stiffness in OA remains unclear, our results generally support the premise that joint stiffness is more related to intraarticular factors, especially synovitis [28].

This study does have some important limitations. It should be noted that our findings should be considered preliminary given the small sample size and the novelty of the assessments performed. A larger sample would enable a more accurate determination of the relative importance of each muscle group we studied in correlating with function. An additional limitation is the cross sectional nature of our study design. We are therefore only able to identify associations between various measures of muscle function and WOMAC scores, but we cannot draw any conclusions about the causality of these relationships. A longitudinal study design would enable us to better determine the predictive value of strength at the hips, knees, and ankles in functional measures in this type of cohort. Finally, because muscle strength at each joint tended to be collinear within individuals, it is possible that strength at each location measured is simply a proxy for a more gross measure of an individual’s strength of the lower limb. While a more robust regression analysis would enable a clearer picture of each muscle group’s importance as an independent predictor of symptoms, our findings of a stronger correlation between WOMAC and muscle function in the quadriceps than other muscle groups suggests at least some degree of independence in the function of these muscles in the symptomatic limb.

## Conclusions

This study found that ultrasound determined muscle thickness had higher levels of measurement reliability than isometric torque testing in multiple muscle groups in the lower limbs of adults with knee OA. Additionally, muscle thickness of the hip abductors, hip adductors, knee extensors and ankle plantarflexors correlates with pain and function but not joint stiffness in adults with symptomatic knee OA. Weaker and thinner muscles in all locations were associated with worse symptoms, and the strongest correlation with symptoms was seen with quadriceps bulk. Future directions for study include a larger sample size to confirm these findings and allow for additional statistical adjustment, as well as a cohort that could be followed longitudinally with repeated strength measures following intervention such as formalized physical therapy. An optimized ultrasound protocol that would be suitable for routine clinical use would be a positive development in evaluating lower limb strength in this population.

## Abbreviations

ACR: American College of Rheumatology
ASIS: Anterior superior iliac spine
ICC: Intraclass correlation coefficients
LEAS: Lower Extremity Activity Scale
OA: Osteoarthritis
WOMAC: Western Ontario and McMaster Universities Arthritis Index
